# Weekly Programming of Hamstring-Related Training Contents in European Professional Soccer

**DOI:** 10.3390/sports12030073

**Published:** 2024-03-05

**Authors:** Pedro Gómez-Piqueras, Antonio Martínez-Serrano, Tomás T. Freitas, Antonio Gómez Díaz, Irineu Loturco, Enric Giménez, Joao Brito, David García-López, Hernan Giuria, Paulino Granero-Gil, Shaun Huygaerts, Francesc Cos, Julio Calleja-González, Emmanuel Vallance, Eduardo Sáez de Villarreal, Pedro E. Alcaraz

**Affiliations:** 1 Paris Saint Germain Football Club, 75016 Paris, France; 2 UCAM Research Center for High Performance Sport, Catholic University of Murcia, 30107 Murcia, Spainpalcaraz@ucam.edu (P.E.A.); 3 Strength and Conditioning Society, 30008 Murcia, Spain; antoniojosegomezdiaz@gmail.com; 4 Faculty of Sport Sciences, Catholic University of Murcia, 30107 Murcia, Spain; 5 NAR—Nucleus of High Performance in Sport, São Paulo 04753-060, Brazil; irineu.loturco@terra.com.br; 6 Spanish Football Federation, 28232 Madrid, Spain; 7 Sports Science Department Sporting FC, San José WVV9+3WR, Costa Rica; enricgimenezmartinez@gmail.com; 8 Portugal Football School, Portuguese Football Federation, 1495-433 Oeiras, Portugal; 9 Department of Health Sciences, European University Miguel de Cervantes, 47012 Valladolid, Spain; dgarcia@uemc.es; 10 Medical Department Club Atlético Rosario Central, Rosario S2000COQ, Argentina; giuriahernan@gmail.com; 11 Ferencváros Torna Club, 1101 Budapest, Hungary; 12 Department of Performance, Royal Antwerp Football Club, 2100 Deurne, Belgium; 13 1st Team, Manchester City Football Club, Manchester M11 3FF, UK; cosfrancesc@gmail.com; 14 Department of Physical Education and Sports, Faculty of Education and Sport, University of the Basque Country, (UPV/EHU), 01007 Vitoria-Gasteiz, Spain; 15 EuroMov Digital Health in Motion, Université de Montpellier, IMT Mines Ales, 34090 Montpellier, France; 16 Physical Performance Sports Research Center (PPSRC), Universidad Pablo de Olavide, 41013 Sevilla, Spain

**Keywords:** football, performance, fatigue, injury, microcycle

## Abstract

Hamstring injuries in soccer continue to be a challenge for professionals who work with soccer players daily. Although its origin is multifactorial, the proper management of neuromuscular fatigue during the training microcycle is a very important factor to consider. There are no clear guidelines regarding the weekly distribution of certain exercises that demand the hamstrings. The main objective of this study was to describe the usual training practices of professional European soccer teams. An international observational survey design was applied to some of the strength and conditioning coaches of professional soccer teams. The survey included different neuromuscular demanding exercises for the hamstrings. For each exercise, the strength and conditioning coaches had to respond in relation to their frequency of use and timepoint depending on the day of the weekly microcycle. Although there is no strong consensus in this regard, there does seem to be a trend when applying certain exercises, especially on the days matchday-4 and matchday-3.

## 1. Introduction

One of the main challenges for a modern soccer team’s performance is how to mitigate the injury risk to increase player availability in training and competition and, consequently, improve team performance. Although injury incidence seems to have decreased in recent years, this trend has not changed in muscle-type injuries [1]. In particular, these types of injuries are frequently located in the hamstring muscles [2], comprising 37% of all muscle injuries and presenting a high recurrence rate of 16% [3]. Despite the efforts made to reduce the injury burden by implementing evidence-based preventive measures, the percentage of muscle injuries, specifically hamstring injuries, unfortunately, has remained constant [4] or even increased [5] over time.

In general, sports injuries are considered to be a very complex and multifactorial phenomenon [6]. Although it is difficult to study the interaction among the different risk factors that cause acute or chronic injuries, there is a general agreement on the fact that these aspects do not act in isolation [7]. For example, among several reasons that could influence the occurrence of hamstring injuries, age, injury history [8], eccentric–concentric strength levels [9], reduced flexibility [10], weekly exposure to high-speed running [11], and neuromuscular fatigue [12] seem to be some of the most important aspects to be considered. This is supported by the fact that hamstring injuries occur more frequently when players are running at high speeds [13], towards the end of the first and second halves of the match, when neuromuscular fatigue levels are at their highest [14,15]. Moreover, another topic worth considering is the load distribution and exercise selection when scheduling preventive programs within the microcycle, given that if not properly implemented, they could also lead to excessive fatigue and be detrimental to performance [16].

To better understand the high incidence of hamstring injuries, it is important to note that the neuromuscular demands placed on this muscle group are high in multiple soccer-specific actions, such as accelerations/decelerations [17], jump landings [18], change of directions, kicking the ball [19], high-speed running, and maximal sprints [20]. Therefore, neuromuscular hamstring fatigue should be optimally managed during training to reduce the risk of injury [21,22]. One of the most determinant factors to consider when implementing injury prevention strategies is the appropriate management of the workload during the training microcycle and pre-season [23].

Nevertheless, the contextual and methodological differences among countries, leagues, and clubs, make it difficult to reach a consensus on the most appropriate way to program training loads and content (e.g., exercise type, volume, intensity, density, and frequency) to minimize injury incidence and to maximize performance [24,25,26]. In this sense, it seems that training loads are higher on match day (MD)-4 and MD-3 of the microcycle, and tend to decrease close to competitions [27,28]. However, the implementation of different types of methodological approaches (i.e., tactical periodization, structured microcycles, periodized or “non-periodized tailored” training programs, etc.) could certainly influence the management and variation of external training load across the annual training season [29,30,31]. This is particularly important for those tasks that, for both performance and injury risk mitigation purposes, involve a greater hamstring neuromuscular demand.

Despite the proven effectiveness of different hamstring exercises in reducing the risk of injury in soccer players [32], it is not clear how these exercises are actually utilized in daily training practices [33,34,35]. Moreover, despite previous research that has already discussed the strategies implemented by practitioners currently working as strength and conditioning coaches (SCCs) in professional soccer teams [36,37,38], a more detailed description of their regular training practices with an emphasis on the exercises that impose high demands on the hamstrings could provide a relevant and concrete information map for soccer practitioners involved in injury mitigation programs. A comprehensive reporting of SCCs’ programming choices can, potentially, add another piece to the puzzle of hamstring injury research by allowing a better understanding of what really happens on a daily basis in real-world contexts. As mentioned above, despite the growing body of evidence concerning hamstring injuries and specific injury mitigation programs, this type of injury continues to rise [5], and one overlooked aspect is related to what is taking place in applied high-performance soccer scenarios. Therefore, the main objective of this study was to describe the habitual training practices of professional European soccer teams based on a survey applied to their respective SCCs, with questions focused on practices concerning the utilization and programming of specific exercises (e.g., stiff-leg deadlift and Nordics) and physical activities (e.g., sprint-oriented and deceleration training) that place high mechanical and neuromuscular demands on the hamstring muscles. Given that the aim of the study was merely descriptive, no leading hypothesis was formulated.

## 2. Materials and Methods

An international observational survey design was applied to a specific cohort (i.e., SCCs of European professional soccer teams). A descriptive design was used for the study. An online survey, including a combination of multiple choices (i.e., only one answer allowed), checkboxes (i.e., multiple answers allowed), and Likert scale responses were used to identify information among practitioners. The questionnaire was designed in a user-friendly manner, with completion only requiring about 15 min. The study followed the recommendations of good practice in questionnaire research [39] and was approved by the local ethics committee (code: CE022106; date: 26 February 2021). Once the objectives of the study had been explained, the SCCs gave their consent to participate in the study. All data were collected and processed anonymously.

A total of 72 professional soccer teams from different European countries (i.e., Belgium, Spain, France, England, Portugal, Russia, and Ukraine) were invited to participate in this study. All invited teams were competing in the first or second division of their countries during the 2020/21 season. Convenience sampling was used as the eligibility criteria due to direct access to the contact of the person in charge of the team’s performance area. The invitation, administered by the International Network of Hamstring Strain Injuries in Football (HSI-Prevent, Consejo Superior de Deportes, Spanish Government), was sent via personal email, which included a link to the survey and explained the main purposes and objectives of the study. Participants were asked to answer the questionnaire and send it back via email. Data were collected between July 2020 and March 2021. Forty-two SCCs (58.3%) (age = 36.8 ± 7.3 years; experience = 9.68 ± 6.3 years) completed the questionnaire. Eighteen SCCs (25%) did not complete the survey correctly, and 12 (16.6%) refused to participate or did not respond to the invitation. The SCCs had to explicitly state their “agreement” or “disagreement” with the future publication of the collected data. All SCCs agreed to this statement.

During April and May 2020, a questionnaire was developed to collect information regarding the prescription and programming of exercises and physical activities that place high mechanical and neuromuscular demands on the hamstrings in professional soccer teams from different European countries. An expert panel comprising some members of the “HSI-Prevent” research group was created to develop the questionnaire. This panel had 18 sport science experts, with extensive experience in soccer performance. Of these, 8 (44.4%) were university professors/researchers, 5 (27.7%) were active as SCCs in elite soccer teams, 3 (16.6%) were sports medicine physicians and 2 (11.1%) were physiotherapists/athletic trainers. Content validity was evaluated by having experts answer the following questions: Were the questions clear and easy to understand? Were the exercises/tasks proposed representative of soccer strength and conditioning practices that place significant demands on hamstring muscles? Would you like the use of this questionnaire for future occasions? Did the questionnaire lack important questions regarding hamstring training practices? Did any of the questions violate your privacy? [40].

After a review of the scientific literature and considering the experience of the soccer experts panel, an initial version of the survey was developed in Spanish. Subsequently, 4 rounds of revision and editing were necessary to produce a pilot version which was unanimously approved by the panel. To ensure face validity (i.e., that all questions were clear and understandable by a representative sample of the collective) this version of the questionnaire was presented to 5 active SCCs that were not part of the initial panel of experts [40], and their comments were implemented in the final version. To improve communication, the questionnaire was translated into English, French, Portuguese, and Russian by respective native speakers that were part of the expert panel. Afterward, a coordinating group composed of 6 researchers (i.e., one for each participating country) was established to contact the different SCCs and distribute the questionnaire. Due to the geographical dispersion of participants and coordinating groups, virtual interaction was used throughout the process for the sake of convenience and efficiency.

The instrument used was a fixed-response questionnaire (the complete version of the questionnaire can be found in Supplementary Material). According to the structured method of training typically used in soccer [41], a total of 26 training activities were grouped as: (1) general tasks (GT); (2) directed tasks (DT); special tasks (ST); and competitive tasks (CT). The different exercises and their respective abbreviations/acronyms are displayed in Table 1. For each task and day of the week about the last game played (MD + 1 and MD + 2), or about the next game to be played (MD-4, MD-3, MD-2, and MD-1), SCCs had to indicate “how many days per week” (i.e., “never [N]—0”, “sometimes [S]—1 to 3”, “frequently [F]—4 to 6”, or “always [A]—6 or more”) and “at which moment” they prescribed the task (i.e., “warm-up/activation [W-U/A]”, “before the session [BF]”, “during the main session [MS]”, “after the session [AFT]”, “before and during the session [BEF-DUR]”, “before and after the session [BEF-AFT]”, and “during and after the session [DUR-AFT]”. While completing the questionnaire, the SCCs of each team were required to respond, considering solely the exercises recommended for players who had a more significant involvement in the most recent game played. It is plausible that players with lesser participation might have had a distinct weekly workload, potentially complicating the interpretation of the results.

Quantitative analysis was used to analyze the questionnaire responses. Incomplete responses were automatically excluded. Statistical analysis was performed using Jamovi® version 1.8.2.0 (Jamovi® project, 2018). Data regarding age and years of experience are presented as means ± standard deviations. A frequency analysis was conducted, with re-sults (all variables) presented as absolute frequency counts and percentages.

## 3. Results

The mean age of the SCCs was 36.8 ± 7.3 years and they had 9.68 ± 6.3 years of experience in professional soccer. Sixteen teams (38.10%) were from France, 15 (35.71%) from Spain, 6 (14.29%) from Portugal, 3 (7.14%) from Russia, 1 from Belgium (2.38%), and 1 (2.38%) from Ukraine. Twenty-seven teams (64.29%) were playing in the first division and 15 (35.71%) in the second division of their countries.

In Table 2 (relative to the first day after the match—MD + 1) and in Table 3 (relative to the second day after the match—MD + 2), a clear trend towards not performing high neuromuscular demand exercises for the hamstrings was found.

**Figure 1:**
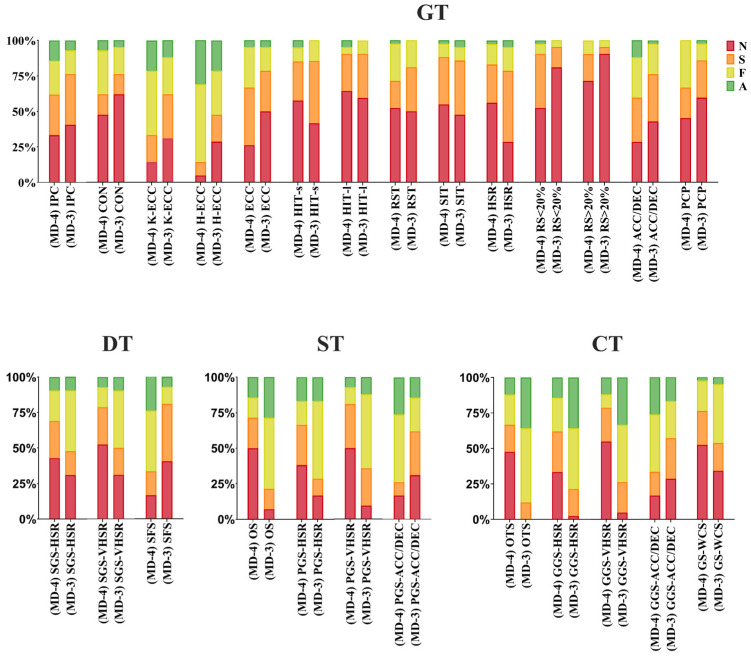
Fourth day before the match (MD-4). ACC/DEC = situations with high intensity and volume of accelerations and decelerations (+2.5 m/s^2^); A = always; CON = high load/volume concentric exercises (>80% RM); CT = competitive tasks; DT = directed tasks; ECC = high-load eccentric exercises (external devices); F = frequently; GGS-ACC/DEC = global game situations with high frequency of accelerations and decelerations (+2.5 m/s^2^); GGS-HSR = global game situations with high-speed running (20–24 km/h); GGS-VHSR = global game situations with very high-speed running (>24 km/h) or >80% Vmax; GS-WCS = game situations reproducing or exceeding the demands of the most demanding scenarios (WCS); GT = general tasks; H-ECC = hip-dominant eccentric exercises; HIT = high-intensity training; HSR = high-speed running (>20 km/h); IPC = isometric posterior chain exercises; K-ECC = knee-dominant eccentric exercises; N = never; OS (>100 m^2^) = opposing situations where the relative area per player is greater than 100 m^2^; OTS (>100 m^2^) = opposing tactical situations where the relative area per player exceeds 100 m^2^; PCP = high-demand posterior chain plyometric exercises; PGS-ACC/DEC = partial game situations with high frequency of high-intensity accelerations and decelerations (+2.5 m/s^2^); PGS-HSR = partial game situations with high-speed running (20–24 km/h); PGS-VHSR = partial game situations with very high-speed running (>24 km/h) or >80% Vmax; RS = resisted-sprint training; RST = repeated-sprint training; SIT = sprint interval training; SFS = specific force situations applied to the game; SGS-HSR = simplified game situations with high-speed running (20–24 km/h); SGS-VHSR = simplified game situations with very high-speed running (>24 km/h); S = sometimes; ST = special tasks.

Regarding the days with the highest general training load for the soccer players (MD-4 and MD-3 to the next match), we noted that most tasks (especially on day MD-3) increased considerably their frequency of use. For both MD-4 and MD-3, this frequency increased more clearly for the group of directed, special, and competitive tasks, and not so much for the group of general tasks (Figure 1).

**Figure 2:**
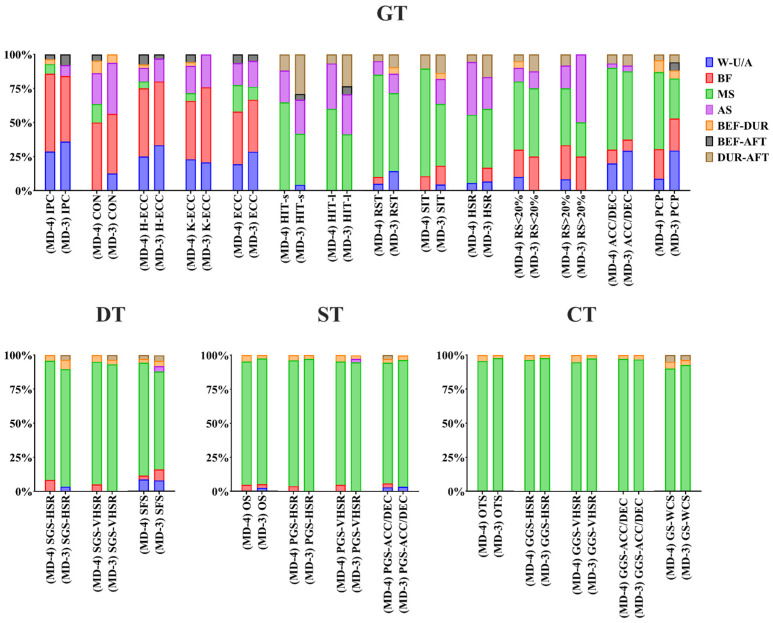
Third day before the match (MD-3). ACC/DEC = situations with high intensity and volume of accelerations and decelerations (+2.5 m/s^2^); AS = after the main session; BEF-AFT = before and after the session; BEF-DUR = before and during the session; BF = before the session; CON = high load/volume concentric exercises (>80% RM); CT = competitive tasks; DT = directed tasks; DUR-AFT = during and after the session; ECC = high-load eccentric exercises (external devices); GGS-ACC/DEC = global game situations with high frequency of accelerations and decelerations (+2.5 m/s^2^); GGS-HSR = global game situations with high-speed running (20–24 km/h); GGS-VHSR = global game situations with very high-speed running (>24 km/h) or >80% Vmax; GS-WCS = game situations reproducing or exceeding the demands of the most demanding scenarios (WCS); GT = general tasks; H-ECC = hip-dominant eccentric exercises; HIT = high-intensity training; HSR = high-speed running (>20 km/h); IPC = isometric posterior chain exercises; K-ECC = knee-dominant eccentric exercises; MS = during the main session; OS (>100 m^2^) = opposing situations where the relative area per player is greater than 100 m^2^; OTS (>100 m^2^) = opposing tactical situations where the relative area per player exceeds 100 m^2^; PCP = high-demand posterior chain plyometric exercises; PGS-ACC/DEC = partial game situations with high frequency of high-intensity accelerations and decelerations (+2.5 m/s^2^); PGS-HSR = partial game situations with high-speed running (20–24 km/h); PGS-VHSR = partial game situations with very high-speed running (>24 km/h) or >80% Vmax; RS = resisted-sprint training; RST = repeated-sprint training; SIT = sprint interval training; SFS = specific force situations applied to the game; SGS-HSR = simplified game situations with high-speed running (20–24 km/h); SGS-VHSR = simplified game situations with very high-speed running (>24 km/h); ST = special tasks; W-U/A = warm-up/activation.

Concerning the time of day in which the tasks were prescribed, we observed a group of general tasks (isometric posterior chain exercises, high load/volume concentric and eccentric exercises, hip- and knee-dominant eccentric exercises), which clearly tended to be prescribed before training or during training warm-up, while the rest (especially those belonging to the groups of directed, special and competitive tasks), were carried out during the main part of the training (Figure 2).

Although two days before the next game (MD-2) the variability was greater in all tasks and all categories, the trend observed in the data indicated that on this day, the neuromuscular demand for the hamstrings was low (Table 4). This trend was similar for MD-1, with the difference that, on this day, it was more common to find global game situations that may have demanded speeds greater than 24 km/h (Table 5).

## 4. Discussion

The proper regulation of weekly workloads within professional soccer teams constitutes a critical aspect to consider due to the requisite metabolic and neuromuscular adaptations. This regulation is essential for achieving an optimal performance state in preparation for subsequent matches [42]. The physical, technical, and tactical contents must be periodized throughout the week considering their accumulated effect on the level of fatigue based on the time until the next match [43,44]. Otherwise, training-induced neuromuscular fatigue, defined as the loss in the ability to generate force [45], may increase the risk of muscle injury [14]. Therefore, the objective of this study was to analyze how the training tasks that are most demanding for the hamstring muscles in soccer teams are distributed during the week. Since muscle injury in this anatomical area has not decreased its incidence in recent years [5], the information presented here could help us to identify, for example, widespread practices that are not recommended. Furthermore, understanding the distribution of training content among professional soccer teams offers valuable insights that SCCs can apply in diverse contexts. The most relevant findings of this study are:

Match day +1 and match day +2

Professional soccer teams typically refrain from engaging in activities with high neuromuscular demands on the hamstrings during the two days subsequent to a match (MD + 1 and MD + 2), across all categories of training (general, directed, special, and competitive). The physiological stress induced by a match results in significant fatigue, necessitating recovery strategies within the following 48 h period [46]. Consequently, it seems coherent to avoid additional efforts that could increase hamstring fatigue, given that this has been identified as a potential risk factor [12]. Nevertheless, it is recommended for players with limited participation in competitive matches to perform such high neuromuscular demand tasks, particularly those involving high-speed activities, to mitigate discrepancies in workload between this group and their more frequently participating counterparts [47].

Match day-4

Four days before the next game (MD-4), teams typically conduct a training session designed to enhance various strength manifestations. This session also aims to facilitate the development of agility, including changes of direction, as well as accelerations and decelerations, by performing drills in small spaces (< 100 m^2^/player) [48]. Although this orientation is common, it does not usually occur in all contexts [28]. A consensus appears to exist regarding the characterization of this session as the most demanding of the week, particularly in terms of external load [24].

On this day of the week, and according to the data collected, we observe that some general tasks are used by most teams (dominant hip and/or knee exercises), or with a tendency to use them (eccentric-overload exercises and situations without the ball where high-intensity accelerations and decelerations predominate). This could be due to the fact that eccentric strength training has been shown to have a protective effect in hamstring strain injuries [49]. Notably, isometrics, high-load concentric exercises, plyometrics, and resisted-sprint training below 20% of body mass present divided opinions in our questionnaire responses. On the contrary, exercises predominantly focused on speed development, such as HIT, RST, SIT, or sled push and pull exercises above 20% of body mass, are typically not incorporated into the training regimen on this day. Consensus appears to be present concerning the timing of these exercises, with the majority of practitioners reporting their execution either before the training or during the main session. In contrast, eccentric exercises involving external resistance represent an area where a significant divergence in opinions emerges regarding the timing of implementation. This question does not seem to be resolved at present by the scientific community either [34,50].

The directed tasks that simulate situations of specific force and the special and competitive tasks where high-intensity accelerations/decelerations appear (partial or global game situations) were used by most of the teams. On the other hand, neither the tasks that stimulate the appearance of high-speed efforts above 24 km/h in its three variants (directed, special, and competitive), nor the special or competitive tasks with a relative space greater than 100 m^2^ / player, nor the competitive dynamics that replicate the “worst case scenarios”, are predominant to this day. This observation suggests that, on this particular day of the week, most of the teams prioritize work in small spaces where very high-speed efforts and sprinting (> 24 km/h) are not developed [51]. Despite this, we found divided opinions regarding the exercises that stimulate high speeds (20–24 km/h), possibly because some teams use intermediate spaces where these values can be easily reached [52]. Notwithstanding these differences, there is a significant agreement regarding the designation of the main part of the session as the appropriate time to develop these training tasks.

Match day-3

Three days before the next game (MD-3), the teams usually hold an endurance-oriented session using large spaces and collective opposition situations. Evidence of this is observed in the day when the distance covered is at its maximum for the entire week [53]. Due to the dimensions of the tasks, distance at high speed is also often a requested variable [52]. This day, together with day MD-4, usually represents the weekly load peak [28].

On this day of the week, most of the general tasks presented in the questionnaire are not commonly used by soccer teams. Although with widely varying frequencies, only high-speed analytical tasks appear to be of significant use on this day. It is likely that the SCCs, aware of the risk of excessive load peaks and fatigue during the week [54,55,56], are likely to limit these more analytical tasks and prioritize more specific situations similar to soccer to achieve sufficient daily load. Furthermore, it is important to highlight that among the teams that do use this type of task, there is a lack of agreement concerning the ideal moment to perform them, especially with concentric and eccentric work, plyometrics, HIT, sled pulls, and pushes.

The directed tasks, where simplified game situations are used, present a greater division of opinions, although with a similar tendency towards non-use. Thus, for example, we find that high-speed or very high-speed situations are used by approximately half of the sample (although with different frequencies). On the contrary, this same type of effort, applied in partial game situations (special tasks) or global (competitive tasks), is used in a significant majority of teams. This, together with the fact that large space tasks (> 100 m^2^/player) are also chosen by the teams on this day, reinforces the previous data found in the literature where it is stated that the total distance and high speed are variables highly stimulated on this day of the week [24,25,28,53].

On the other hand, regarding high-intensity accelerations and decelerations, we noted a significantly broader range of responses. The inter-device variability when collecting this variable [57], the lack of consensus regarding the different methods of measurement [58], and the difficulty of relativizing the variable depending on the initial speed of the players [59], could be creating confusion or disparity of concepts among the SCCs when answering this question. Similarly, we also found a considerable variability in determining the importance of exposing the player to work-case scenarios on this day of the week. It is possible that due to the novelty of this concept and its still imprecise definition/measurement [60], the SCCs have employed specific methods for its categorization.

Match day-2

Two days before the next game (MD-2), some methodologies like tactical periodization prioritize speed and sprint efforts [61]. This vision is not shared by other methodologies that prefer to use recovery and tapering strategies on this day of the week to reach an optimal state for the competition [28]. Consequently, the distance covered at high speeds and through sprinting during the week, despite being crucial for enhancing performance and mitigating the risk of hamstring injuries [62], exhibits a different weekly periodization depending on the methodology used. Regardless of the approach, it would be beneficial for the player to reach high percentages of maximum speed (> 85%) in one of these training sessions, since this could reduce the risk of muscle injury in the hamstrings [35,63].

Based on our data, we observe that most of the teams analyzed do not perform any of the general, directed, special, or competitive tasks presented during this day of the week. Only high speeds (20–24 km/h) in partial game situations showed greater variability. Sprint training is also usually carried out by most teams during MD-3 and not MD-2, as tactical periodization recommends.

Match day-1

One day before the next game (MD-1), soccer teams typically undertake a session characterized by a light workload. This session aims to optimize players’ preparation for the upcoming competition, during which the external load values, as measured by GPS, are generally observed to be the lowest throughout the week [26]. The physical component is not usually a priority on this day, and tactical and emotional issues are prioritized.

Although certain general and analytical tasks outlined in the questionnaire, such as concentric work, plyometrics, and short sprints, may contribute positively to subsequent performance [64], the majority of teams reported abstaining from these activities on the day preceding the competition. Something similar can be observed with the directed, special, and competitive tasks, which also do not present significant frequencies of use. Only global game situations (competitive), where relative surfaces greater than 100 m^2^/player were used and where high speeds (20–24 km/h) were stimulated, present higher frequencies of use. These tasks could correspond to game situations where the coach tries to remember the collective behaviors to prioritize for the next day’s game.

Limitations

There are certain limitations when evaluating the weekly content periodization of soccer teams through our questionnaire. In the first place, the samples of the different competitions were not similar (level or games per week). This could condition the answers if we consider that, in certain countries, the SCCs are more likely to use one methodology than another. Secondly, there are important and representative top countries of professional soccer, such as England or Germany, which did not participate in this study. Thirdly, dynamic or static stretching exercises were not included in the questionnaire as part of the warm-up or cool-down strategies, despite their potential effects for hamstring injury prevention [65,66]. Furthermore, in order to identify the effectiveness of the exercises and programming strategies used, it would have been valuable to collect information of the injuries sustained by the team during the development of the study. However, such data were not made available by every club/SCC involved, so this analysis was not possible. Lastly, even though the questionnaire was created by a group of experts with extensive knowledge of soccer training, it is possible that some SCCs do not use a similar nomenclature when cataloging the different training exercises and their corresponding load variables. This could create ambiguity and confusion when answering some of the questions raised.

## 5. Conclusions

The present study described, in detail, the weekly programming choices of professional soccer SCCs as they relate to the different types of tasks/exercises that specifically target the hamstring muscles. Moreover, to our knowledge, the current research addressed, for the first time, how the varied contents are placed throughout the in-season microcyle, having the game as reference (i.e., we identify the practices according to match day: MD + 1, MD + 2, MD-4, etc.) in European professional soccer.

In summary, during the two days following the game (MD + 1 and MD + 2), teams do not usually use high neuromuscular demand tasks for the hamstrings. Four days before the next match (MD-4), the tasks that simulate specific force situations and those where high-intensity accelerations and decelerations appear frequently are used by the majority of practitioners. On the other hand, during this day the teams do not use speed-related tasks. Three days before the game (MD-3) is when the greatest discrepancies amongst practitioners were identified. Nevertheless, in general, it appears that the SCCs prefer to expose their players to wide spaces where high or very high speeds are requested. The use of more analytical tasks before this training session is not common. Finally, during the two days before the game (MD-2 and MD-1), the teams do not seem to have the habit of using demanding tasks for the hamstring muscles in any of their variants and/or moments. Considering that the incidence of hamstring injuries is still amongst the highest in professional soccer, the present findings could be used as a starting point to analyze if current practices are effective to reduce injury burdens in this muscle group. From a practical perspective, the lack of agreement regarding MD-3 programming in terms of whether or not (or when) to perform specific exercises (e.g., eccentric-based) indicates that further research is still needed regarding the effects of different microcycle designs to mitigate hamstring injuries.

## Figures and Tables

**Table 1:** Different exercises and their abbreviations used within each type of training tasks.

Type	Tasks	Abbreviature
General Tasks (GT)	Isometric posterior chain exercises.	IPC
	High load/volume concentric exercises (>80% RM).	CON
	Hip-dominant eccentric exercises.	H-ECC
	Knee-dominant eccentric exercises.	K-ECC
	High-load eccentric exercises (external devices).	ECC
	High-intensity training (100–120% vVO_2max_).	HIT
	High-intensity training (90–100% vVO_2max_).	
	Repeated-sprint training (70–85% V_max_).	RST
	Sprint interval training (85–100% V_max_).	SIT
	High-speed running (>20 km/h).	HSR
	Resisted-sprint training (<20% BM).	RS
	Resisted-sprint training (>20% BM).	
	Situations with high intensity and volume of accelerations and decelerations (+2.5 m/s^2^).	ACC/DEC
	High-demand posterior chain plyometric exercises.	PCP
Directed Tasks (DT)	Simplified game situations with high-speed running (20–24 km/h).	SGS-HSR
	Simplified game situations with very high-speed running (>24 km/h).	SGS-VHSR
	Specific strength situations applied to the game.	SFS
Special Tasks (ST)	Opposing situations where the relative area per player is greater than 100 m^2^.	OS (>100 m^2^)
	Partial game situations with high-speed running (20–24 km/h).	PGS-HSR
	Partial game situations with very high-speed running (>24 km/h) or >80% V_max_.	PGS-VHSR
	Partial game situations with high frequency of high-intensity accelerations and decelerations (+2.5 m/s^2^).	PGS-ACC/DEC
Competitive Tasks (CT)	Opposing tactical situations where the relative area per player exceeds 100 m^2^.	OTS (>100 m^2^)
	Global game situations with high-speed running (20–24 km/h).	GGS-HSR
	Global game situations with very high-speed running (>24 km/h) or >80% V_max_.	GGS-VHSR
	Global game situations with high frequency of accelerations and decelerations (+2.5 m/s^2^).	GGS-ACC/DEC
	Game situations reproducing or exceeding the demands of the most demanding scenarios.	GS-WCS

**Table 2:** First day after the match (MD + 1).

	Type of Task	Frequency [*n* (%)]				Moment [*n* (%)]						
		N	S	F	A	W-U/A	BF	MS	AS	BEF-DUR	BEF-AFT	DUR-AFT
GT	IPC	**28 (66.7)**	9 (21.4)	3 (7.1)	2 (4.8)	6 (42.9)	6 (42.9)	1 (7.1)	0 (0.0)	1 (7.1)	0 (0.0)	0 (0.0)
	CON (>80% RM)	**34 (81.0)**	4 (9.5)	4 (9.5)	0 (0.0)	0 (0.0)	4 (50.0)	2 (25.0)	1 (12.5)	1 (12.5)	0 (0.0)	0 (0.0)
	H-ECC	17 (40.5)	12 (28.6)	8 (19.0)	5 (11.9)	9 (36.0)	12 (48.0)	1 (4.0)	1 (4.0)	2 (8.0)	0 (0.0)	0 (0.0)
	K-ECC	**22 (52.4)**	9 (21.4)	8 (19.0)	3 (7.1)	5 (25.0)	11 (55.0)	0 (0.0)	3 (15.0)	1 (5.0)	0 (0.0)	0 (0.0)
	ECC (external devices)	**25 (59.5)**	12 (28.6)	4 (9.5)	1 (2.4)	4 (23.5)	7 (41.2)	4 (23.5)	1 (5.9)	0 (0.0)	1 (5.9)	0 (0.0)
	HIT (100–120% vVO_2max_)	19 (46.3)	12 (29.3)	8 (19.5)	2 (4.9)	0 (0.0)	0 (0.0)	14 (60.9)	4 (17.4)	0 (0.0)	0 (0.0)	5 (21.7)
	HIT (90–100% vVO_2max_)	**28 (68.3)**	10 (24.4)	2 (4.9)	1 (2.4)	0 (0.0)	2 (14.3)	7 (50.0)	2 (14.3)	0 (0.0)	0 (0.0)	3 (21.4)
	RST (70–85% V_max_)	**23 (54.8)**	10 (23.8)	8 (19.0)	1 (2.4)	0 (0.0)	0 (0.0)	15 (78.9)	1 (5.3)	0 (0.0)	0 (0.0)	3 (15.8)
	SIT (85–100% V_max_)	**25 (59.5)**	11 (26.2)	5 (11.9)	1 (2.4)	0 (0.0)	1 (5.9)	16 (94.1)	0 (0.0)	0 (0.0)	0 (0.0)	0 (0.0)
	HSR	**23 (56.1)**	11 (26.8)	7 (17.1)	0 (0.0)	0 (0.0)	1 (5.9)	8 (47.1)	5 (29.4)	1 (5.9)	0 (0.0)	2 (11.8)
	RS (<20% BM)	**28 (66.7)**	13 (31.0)	1 (2.4)	0 (0.0)	3 (21.4)	1 (7.1)	6 (42.9)	1 (7.1)	1 (7.1)	0 (0.0)	2 (14.3)
	RS (>20% BM)	**35 (83.3)**	7 (16.7)	0 (0.0)	0 (0.0)	1 (14.3))	2 (28.6)	3 (42.9)	1 (14.3)	0 (0.0)	0 (0.0)	0 (0.0)
	ACC/DEC	**22 (52.4)**	13 (31.0)	7 (16.7)	0 (0.0)	3 (15.0)	0 (0.0)	13 (65.0)	0 (0.0)	0 (0.0)	1 (5.0)	3 (15.0)
	PCP	**25 (59.5)**	16 (38.1)	1 (2.4)	0 (0.0)	2 (11.8)	3 (17.6)	8 (47.1)	0 (0.0)	2 (11.8)	0 (0.0)	2 (11.8)
DT	SGS-HSR	19 (45.2)	4 (9.5)	15 (35.7)	4 (9.5)	0 (0.0)	0 (0.0)	22 (95.7)	0 (0.0)	1 (4.3)	0 (0.0)	0 (0.0)
	SGS-VHSR	17 (40.5)	9 (21.4)	14 (33.3)	2 (4.8)	0 (0.0)	0 (0.0)	24 (96.0)	0 (0.0)	1 (4.0)	0 (0.0)	0 (0.0)
	SFS	18 (42.9)	6 (14.3)	15 (35.7)	3 (7.1)	2 (8.3)	0 (0.0)	22 (91.7)	0 (0.0)	0 (0.0)	0 (0.0)	0 (0.0)
ST	OS (>100 m^2^)	**26 (61.9)**	7 (16.7)	7 (16.7)	2 (4.8)	0 (0.0)	0 (0.0)	15 (93.8)	0 (0.0)	1 (6.3)	0 (0.0)	0 (0.0)
	PGS-HSR	**24 (57.1)**	8 (19.0)	9 (21.4)	1 (2.4)	0 (0.0)	0 (0.0)	17 (94.4)	0 (0.0)	1 (5.6)	0 (0.0)	0 (0.0)
	PGS-VHSR	**26 (61.9)**	7 (16.7)	8 (19.0)	1 (2.4)	0 (0.0)	0 (0.0)	15 (93.8)	0 (0.0)	1 (6.3)	0 (0.0)	0 (0.0)
	PGS-ACC/DEC	17 (40.5)	6 (14.3)	15 (35.7)	4 (9.5)	1 (4.0)	0 (0.0)	22 (88.0)	0 (0.0)	1 (4.0)	0 (0.0)	1 (4.0)
CT	OTS (>100 m^2^)	**28 (66.7)**	4 (9.5)	5 (11.9)	5 (11.9)	0 (0.0)	0 (0.0)	13 (92.9)	0 (0.0)	0 (0.0)	0 (0.0)	1 (7.1)
	GGS-HSR	**23 (54.8)**	8 (19.0)	7 (16.7)	4 (9.5)	0 (0.0)	0 (0.0)	18 (94.7)	0 (0.0)	1 (5.3)	0 (0.0)	0 (0.0)
	GGS-VHSR	**23 (57.5)**	10 (25.0)	4 (10.0)	3 (7.5)	0 (0.0)	0 (0.0)	15 (88.2)	0 (0.0)	1 (5.9)	0 (0.0)	1 (5.9)
	GGS-ACC/DEC	19 (45.2)	5 (11.9)	11 (26.2)	7 (16.7)	0 (0.0)	0 (0.0)	22 (95.7)	0 (0.0)	1 (4.3)	0 (0.0)	0 (0.0)
	GS-WCS	**25 (59.5)**	0 (0.0)	6 (14.3)	11 (26.2)	0 (0.0)	0 (0.0)	17 (100.0)	0 (0.0)	0 (0.0)	0 (0.0)	0 (0.0)

Bold numbers represent frequencies > 50%. ACC/DEC = situations with high intensity and volume of accelerations and decelerations (+2.5 m/s^2^); CON = high load/volume concentric exercises (>80%RM); CT = competitive tasks; DT = directed tasks; ECC = high-load eccentric exercises (external devices); GGS-ACC/DEC = global game situations with high frequency of accelerations and decelerations (+2.5 m/s^2^); GGS-HSR = global game situations with high-speed running (20–24 km/h); GGS-VHSR = global game situations with very high-speed running (>24 km/h) or >80% V_max_; GS-WCS = game situations reproducing or exceeding the demands of the most demanding scenarios (WCS); GT = general tasks; H-ECC = hip-dominant eccentric exercises; HIT = high-intensity training; HSR = high-speed running (>20 km/h); IPC = isometric posterior chain exercises; K-ECC = knee-dominant eccentric exercises; OS (>100 m^2^) = opposing situations where the relative area per player is greater than 100 m^2^; OTS (>100 m^2^) = opposing tactical situations where the relative area per player exceeds 100 m^2^; PCP = high-demand posterior chain plyometric exercises; PGS-ACC/DEC = partial game situations with high frequency of high-intensity accelerations and decelerations (+2.5 m/s^2^); PGS-HSR = partial game situations with high-speed running (20–24 km/h); PGS-VHSR = partial game situations with very high-speed running (>24 km/h) or >80% V_max_; RS = resisted-sprint training; RST = repeated-sprint training; SIT = sprint interval training; SFS = specific force situations applied to the game; SGS-HSR = simplified game situations with high-speed running (20–24 km/h); SGS-VHSR = simplified game situations with very high-speed running (>24 km/h); ST = special tasks.

**Table 3:** Second day after the match (MD + 2).

	Type of Task	Frequency [*n* (%)]				Moment [*n* (%)]						
		N	S	F	A	W-U/A	BF	MS	AS	BEF-DUR	BEF-AFT	DUR-AFT
GT	IPC	**31 (73.8)**	1 (2.4)	0 (0.0)	10 (23.8)	4 (36.4)	6 (54.5)	1 (9.1)	0 (0.0)	0 (0.0)	0 (0.0)	0 (0.0)
	CON (>80% RM)	**37 (88.1)**	4 (9.5)	1 (2.4)	0 (0.0)	0 (0.0)	2 (40.0)	0 (0.0)	3 (60.0)	0 (0.0)	0 (0.0)	0 (0.0)
	H-ECC	**28 (66.7)**	7 (16.7)	5 (11.9)	2 (4.8)	2 (14.3)	6 (42.9)	1 (7.1)	5 (35.7)	0 (0.0)	0 (0.0)	0 (0.0)
	K-ECC	**30 (73.2)**	6 (14.6)	4 (9.8)	1 (2.4)	2 (18.2)	4 (36.4)	1 (9.1)	4 (36.4)	1 (5.0)	0 (0.0)	0 (0.0)
	ECC (external devices)	**33 (80.5)**	6 (14.6)	1 (2.4)	1 (2.4)	0 (0.0)	3 (37.5)	2 (25.0)	3 (37.5)	0 (0.0)	0 (0.0)	0 (0.0)
	HIT (100–120% vVO_2max_)	**31 (73.8)**	8 (19.0)	3 (7.1)	0 (0.0)	0 (0.0)	1 (9.1)	7 (63.6)	2 (18.2)	0 (0.0)	0 (0.0)	1 (9.1)
	HIT (90–100% vVO_2max_)	**33 (78.6)**	7 (16.7)	2 (4.8)	0 (0.0)	0 (0.0)	0 (0.0)	7 (77.8)	1 (11.1)	0 (0.0)	0 (0.0)	1 (11.1)
	RST (70–85% V_max_)	**34 (81.0)**	4 (9.5)	3 (7.1)	1 (2.4)	0 (0.0)	0 (0.0)	6 (75.0)	1 (12.5)	1 (12.5)	0 (0.0)	0 (0.0)
	SIT (85–100% V_max_)	**34 (81.0)**	6 (14.3)	2 (4.8)	0 (0.0)	1 (12.5)	0 (0.0)	6 (75.0)	1 (12.5)	0 (0.0)	0 (0.0)	0 (0.0)
	HSR	**31 (73.8)**	8 (19.0)	2 (4.8)	1 (2.4)	0 (0.0)	0 (0.0)	7 (63.6)	3 (27.3)	0 (0.0)	0 (0.0)	1 (9.1)
	RS (<20% BM)	**38 (90.5)**	3 (7.1)	1 (2.4)	0 (0.0)	0 (0.0)	2 (50.0)	1 (25.0)	1 (25.0)	0 (0.0)	0 (0.0)	0 (0.0)
	RS (>20% BM)	**40 (95.2)**	1 (2.4)	1 (2.4)	0 (0.0)	0 (0.0)	2 (100.0)	0 (0.0)	0 (0.0)	0 (0.0)	0 (0.0)	0 (0.0)
	ACC/DEC	**31 (73.8)**	5 (11.9)	5 (11.9)	1 (2.4)	2 (18.2)	0 (0.0)	9 (81.8)	0 (0.0)	0 (0.0)	0 (0.0)	0 (0.0)
	PCP	**36 (85.7)**	4 (9.5)	2 (4.8)	0 (0.0)	2 (33.3)	1 (16.7)	3 (50.0)	0 (0.0)	0 (0.0)	0 (0.0)	0 (0.0)
DT	SGS-HSR	**29 (69.0)**	6 (14.3)	7 (16.7)	0 (0.0)	1 (7.7)	0 (0.0)	9 (69.2)	2 (15.4)	0 (0.0)	0 (0.0)	1 (7.7)
	SGS-VHSR	**32 (76.2)**	7 (16.7)	3 (7.1)	0 (0.0)	0 (0.0)	0 (0.0)	8 (80.0)	2 (20.0)	0 (0.0)	0 (0.0)	0 (0.0)
	SFS	**27 (64.3)**	8 (19.0)	7 (16.7)	0 (0.0)	1 (6.7)	0 (0.0)	12 (80.0)	2 (13.3)	0 (0.0)	0 (0.0)	0 (0.0)
ST	OS (>100 m^2^)	**33 (78.6)**	1 (2.4)	8 (19.0)	0 (0.0)	0 (0.0)	0 (0.0)	8 (88.9)	1 (11.1)	0 (0.0)	0 (0.0)	0 (0.0)
	PGS-HSR	**32 (76.2)**	5 (11.9)	4 (9.5)	1 (2.4)	0 (0.0)	0 (0.0)	10 (100.0)	0 (0.0)	0 (0.0)	0 (0.0)	0 (0.0)
	PGS-VHSR	**36 (85.7)**	4 (9.5)	2 (4.8)	0 (0.0)	0 (0.0)	0 (0.0)	6 (100.0)	0 (0.0)	0 (0.0)	0 (0.0)	0 (0.0)
	PGS-ACC/DEC	**27 (64.3)**	4 (9.5)	10 (23.8)	1 (2.4)	0 (0.0)	0 (0.0)	15 (100.0)	0 (0.0)	0 (0.0)	0 (0.0)	0 (0.0)
CT	OTS (>100 m^2^)	**32 (76.2)**	5 (11.9)	4 (9.5)	1 (2.4)	0 (0.0)	0 (0.0)	9 (90.0)	1 (10.0)	0 (0.0)	0 (0.0)	0 (0.0)
	GGS-HSR	**32 (76.2)**	6 (14.3)	3 (7.1)	1 (2.4)	0 (0.0)	0 (0.0)	9 (90.0)	1 (10.0)	0 (0.0)	0 (0.0)	0 (0.0)
	GGS-VHSR	**33 (78.6)**	6 (14.3)	2 (4.8)	1 (2.4)	0 (0.0)	0 (0.0)	8 (88.9)	1 (11.1)	0 (0.0)	0 (0.0)	0 (0.0)
	GGS-ACC/DEC	**27 (64.3)**	5 (11.9)	7 (16.7)	3 (7.1)	0 (0.0)	0 (0.0)	14 (93.3)	1 (6.7)	0 (0.0)	0 (0.0)	0 (0.0)
	GS-WCS	**35 (85.4)**	5 (12.2)	1 (2.4)	0 (0.0)	0 (0.0)	0 (0.0)	5 (83.3)	1 (16.7)	0 (0.0)	0 (0.0)	0 (0.0)

Bold numbers represent frequencies > 50%. ACC/DEC = situations with high intensity and volume of accelerations and decelerations (+2.5 m/s^2^); CON = high load/volume concentric exercises (>80%RM); CT = competitive tasks; DT = directed tasks; ECC = high-load eccentric exercises (external devices); GGS-ACC/DEC = global game situations with high frequency of accelerations and decelerations (+2.5 m/s^2^); GGS-HSR = global game situations with high-speed running (20–24 km/h); GGS-VHSR = global game situations with very high-speed running (>24 km/h) or > 80% V_max_; GS-WCS = game situations reproducing or exceeding the demands of the most demanding scenarios (WCS); GT = general tasks; H-ECC = hip-dominant eccentric exercises; HIT = high-intensity training; HSR = high-speed running (>20 km/h); IPC = isometric posterior chain exercises; K-ECC = knee-dominant eccentric exercises; OS (>100 m^2^) = opposing situations where the relative area per player is greater than 100 m^2^; OTS (>100 m^2^) = opposing tactical situations where the relative area per player exceeds 100 m^2^; PCP = high-demand posterior chain plyometric exercises; PGS-ACC/DEC = partial game situations with high frequency of high-intensity accelerations and decelerations (+2.5 m/s^2^); PGS-HSR = partial game situations with high-speed running (20–24 km/h); PGS-VHSR = partial game situations with very high-speed running (>24 km/h) or >80% V_max_; RS = resisted-sprint training; RST = repeated-sprint training; SIT = sprint interval training; SFS = specific force situations applied to the game; SGS-HSR = simplified game situations with high-speed running (20–24 km/h); SGS-VHSR = simplified game situations with very high-speed running (>24 km/h); ST = special tasks.

**Table 4:** Second day before the match (MD-2).

	Type of Task	Frequency [*n* (%)]				Moment [*n* (%)]						
		N	S	F	A	W-U/A	BF	MS	AS	BEF-DUR	BEF-AFT	DUR-AFT
GT	IPC	**21 (52.5)**	7 (17.5)	9 (22.5)	3 (7.5)	9 (47.4)	8 (42.1)	0 (0.0)	0 (0.0)	0 (0.0)	2 (10.5)	0 (0.0)
	CON (>80% RM)	**37 (88.1)**	4 (9.5)	1 (2.4)	0 (0.0)	0 (0.0)	3 (60.0)	0 (0.0)	2 (40.0)	0 (0.0)	0 (0.0)	0 (0.0)
	H-ECC	**23 (54.8)**	10 (23.8)	5 (11.9)	4 (9.5)	8 (42.1)	9 (47.4)	0 (0.0)	2 (10.5)	0 (0.0)	0 (0.0)	0 (0.0)
	K-ECC	**27 (64.3)**	9 (21.4)	4 (9.5)	2 (4.8)	3 (20.0)	10 (66.7)	0 (0.0)	2 (13.3)	0 (0.0)	0 (0.0)	0 (0.0)
	ECC (external devices)	**37 (88.1)**	3 (7.1)	2 (4.8)	0 (0.0)	1 (20.0)	3 (60.0)	0 (0.0)	1 (20.0)	0 (0.0)	0 (0.0)	0 (0.0)
	HIT (100–120% vVO_2max_)	**40 (95.2)**	1 (2.4)	1 (2.4)	0 (0.0)	0 (0.0)	0 (0.0)	1 (50.0)	1 (50.0)	0 (0.0)	0 (0.0)	0 (0.0)
	HIT (90–100% vVO_2max_)	**41 (97.6)**	1 (2.4)	0 (0.0)	0 (0.0)	0 (0.0)	0 (0.0)	1 (100.0)	0 (0.0)	0 (0.0)	0 (0.0)	0 (0.0)
	RST (70–85% V_max_)	**38 (90.5)**	2 (4.8)	2 (4.8)	0 (0.0)	2 (50.0)	0 (0.0)	1 (25.0)	0 (0.0)	0 (0.0)	0 (0.0)	1 (25.0)
	SIT (85–100% V_max_)	**38 (92.7)**	0 (0.0)	3 (7.3)	0 (0.0)	2 (66.7)	0 (0.0)	1 (33.3)	0 (0.0)	0 (0.0)	0 (0.0)	0 (0.0)
	HSR	**33 (82.5)**	3 (7.5)	3 (7.5)	1 (2.5)	0 (0.0)	0 (0.0)	4 (57.1)	1 (14.3)	0 (0.0)	0 (0.0)	2 (28.6)
	RS (<20% BM)	**39 (92.9)**	2 (4.8)	0 (0.0)	1 (2.4)	0 (0.0)	1 (33.3)	1 (33.3)	0 (0.0)	0 (0.0)	1 (33.3)	0 (0.0)
	RS (>20% BM)	**41 (97.6)**	1 (2.4)	0 (0.0)	0 (0.0)	1 (100.0)	0 (0.0)	0 (0.0)	0 (0.0)	0 (0.0)	0 (0.0)	0 (0.0)
	ACC/DEC	**36 (87.8)**	4 (9.8)	1 (2.4)	0 (0.0)	4 (80.0)	0 (0.0)	1 (20.0)	0 (0.0)	0 (0.0)	0 (0.0)	0 (0.0)
	PCP	**37 (88.1)**	2 (4.8)	3 (7.1)	0 (0.0)	3 (60.0)	1 (20.0)	0 (0.0)	0 (0.0)	0 (0.0)	1 (20.0)	0 (0.0)
DT	SGS-HSR	**24 (57.1)**	8 (19.0)	6 (14.3)	4 (9.5)	2 (11.1)	0 (0.0)	15 (83.3)	0 (0.0)	1 (5.6)	0 (0.0)	0 (0.0)
	SGS-VHSR	**27 (64.3)**	7 (16.7)	7 (16.7)	1 (2.4)	0 (0.0)	1 (6.7)	13 (86.7)	0 (0.0)	1 (6.7)	0 (0.0)	0 (0.0)
	SFS	**32 (76.2)**	7 (16.7)	2 (4.8)	1 (2.4)	3 (30.0)	0 (0.0)	7 (70.0)	0 (0.0)	0 (0.0)	0 (0.0)	0 (0.0)
ST	OS (>100 m^2^)	**27 (64.3)**	7 (16.7)	7 (16.7)	1 (2.4)	0 (0.0)	0 (0.0)	15 (100.0)	0 (0.0)	0 (0.0)	0 (0.0)	0 (0.0)
	PGS-HSR	17 (40.5)	14 (33.3)	9 (21.4)	2 (4.8)	0 (0.0)	0 (0.0)	24 (96.0)	0 (0.0)	1 (4.0)	0 (0.0)	0 (0.0)
	PGS-VHSR	**30 (71.4)**	5 (11.9)	5 (11.9)	2 (4.8)	0 (0.0)	0 (0.0)	11 (91.7)	0 (0.0)	1 (8.3)	0 (0.0)	0 (0.0)
	PGS-ACC/DEC	**32 (76.2)**	6 (14.3)	4 (9.5)	0 (0.0)	0 (0.0)	0 (0.0)	10 (100.0)	0 (0.0)	0 (0.0)	0 (0.0)	0 (0.0)
CT	OTS (>100 m^2^)	**26 (61.9)**	11 (26.2)	3 (7.1)	2 (4.8)	0 (0.0)	0 (0.0)	16 (100.0)	0 (0.0)	0 (0.0)	0 (0.0)	0 (0.0)
	GGS-HSR	**31 (73.8)**	7 (16.7)	1 (2.4)	3 (7.1)	0 (0.0)	0 (0.0)	10 (90.9)	0 (0.0)	1 (9.1)	0 (0.0)	0 (0.0)
	GGS-VHSR	**35 (83.3)**	4 (9.5)	1 (2.4)	2 (4.8)	0 (0.0)	0 (0.0)	6 (85.7)	0 (0.0)	1 (14.3)	0 (0.0)	0 (0.0)
	GGS-ACC/DEC	**32 (76.2)**	8 (19.0)	2 (4.8)	0 (0.0)	0 (0.0)	0 (0.0)	10 (100.0)	0 (0.0)	0 (0.0)	0 (0.0)	0 (0.0)
	GS-WCS	**40 (95.2)**	2 (4.8)	0 (0.0)	0 (0.0)	0 (0.0)	0 (0.0)	2 (100.0)	0 (0.0)	0 (0.0)	0 (0.0)	0 (0.0)

Bold numbers represent frequencies > 50%. ACC/DEC = situations with high intensity and volume of accelerations and decelerations (+2.5 m/s^2^); CON = high load/volume concentric exercises (>80%RM); CT = competitive tasks; DT = directed tasks; ECC = high-load eccentric exercises (external devices); GGS-ACC/DEC = global game situations with high frequency of accelerations and decelerations (+2.5 m/s^2^); GGS-HSR = global game situations with high-speed running (20–24 km/h); GGS-VHSR = global game situations with very high-speed running (>24 km/h) or >80% V_max_; GS-WCS = game situations reproducing or exceeding the demands of the most demanding scenarios (WCS); GT = general tasks; H-ECC = hip-dominant eccentric exercises; HIT = high-intensity training; HSR = high-speed running (>20 km/h); IPC = isometric posterior chain exercises; K-ECC = knee-dominant eccentric exercises; OS (>100 m^2^) = opposing situations where the relative area per player is greater than 100 m^2^; OTS (>100 m^2^) = opposing tactical situations where the relative area per player exceeds 100 m^2^; PCP = high-demand posterior chain plyometric exercises; PGS-ACC/DEC = partial game situations with high frequency of high-intensity accelerations and decelerations (+2.5 m/s^2^); PGS-HSR = partial game situations with high-speed running (20–24 km/h); PGS-VHSR = partial game situations with very high-speed running (>24 km/h) or >80% V_max_; RS = resisted-sprint training; RST = repeated-sprint training; SIT = sprint interval training; SFS = specific force situations applied to the game; SGS-HSR = simplified game situations with high-speed running (20–24 km/h); SGS-VHSR = simplified game situations with very high-speed running (>24 km/h); ST = special tasks.

**Table 5:** One day before the match (MD-1).

	Type of Task	Frequency [*n* (%)]				Moment [*n* (%)]						
		N	S	F	A	W-U/A	BF	MS	AS	BEF-DUR	BEF-AFT	DUR-AFT
GT	IPC	**31 (73.8)**	7 (16.7)	4 (9.5)	0 (0.0)	6 (54.5)	5 (45.5)	0 (0.0)	0 (0.0)	0 (0.0)	0 (0.0)	0 (0.0)
	CON (>80% RM)	**41 (97.6)**	1 (2.4)	0 (0.0)	0 (0.0)	0 (0.0)	1 (100.0)	0 (0.0)	0 (0.0)	0 (0.0)	0 (0.0)	0 (0.0)
	H-ECC	**30 (71.4)**	7 (16.7)	3 (7.1)	2 (4.8)	6 (50.0)	6 (50.0)	0 (0.0)	0 (0.0)	0 (0.0)	0 (0.0)	0 (0.0)
	K-ECC	**36 (85.7)**	5 (11.9)	1 (2.4)	0 (0.0)	1 (16.7)	4 (66.7)	0 (0.0)	1 (16.7)	0 (0.0)	0 (0.0)	0 (0.0)
	ECC (external devices)	**41 (97.6)**	0 (0.0)	1 (2.4)	0 (0.0)	1 (100.0)	0 (0.0)	0 (0.0)	0 (0.0)	0 (0.0)	0 (0.0)	0 (0.0)
	HIT (100–120% vVO_2max_)	**41 (97.6)**	1 (2.4)	0 (0.0)	0 (0.0)	0 (0.0)	0 (0.0)	1 (100.0)	0 (0.0)	0 (0.0)	0 (0.0)	0 (0.0)
	HIT (90–100% vVO_2max_)	**41 (97.6)**	1 (2.4)	0 (0.0)	0 (0.0)	0 (0.0)	0 (0.0)	1 (100.0)	0 (0.0)	0 (0.0)	0 (0.0)	0 (0.0)
	RST (70–85% V_max_)	**38 (90.5)**	2 (4.8)	2 (4.8)	0 (0.0)	0 (0.0)	1 (25.0)	3 (75.0)	0 (0.0)	0 (0.0)	0 (0.0)	0 (0.0)
	SIT (85–100% V_max_)	**36 (85.7)**	2 (4.8)	2 (4.8)	2 (4.8)	1 (16.7)	1 (16.7)	4 (66.7)	0 (0.0)	0 (0.0)	0 (0.0)	0 (0.0)
	HSR	**39 (92.9)**	2 (4.8)	1 (2.4)	0 (0.0)	1 (33.3)	1 (33.3)	1 (33.3)	0 (0.0)	0 (0.0)	0 (0.0)	0 (0.0)
	RS (<20% BM)	**42 (100.0)**	0 (0.0)	0 (0.0)	0 (0.0)	0 (0.0)	0 (0.0)	0 (0.0)	0 (0.0)	0 (0.0)	0 (0.0)	0 (0.0)
	RS (>20% BM)	**42 (100.0)**	0 (0.0)	0 (0.0)	0 (0.0)	0 (0.0)	0 (0.0)	0 (0.0)	0 (0.0)	0 (0.0)	0 (0.0)	0 (0.0)
	ACC/DEC	**27 (64.3)**	5 (11.9)	7 (16.7)	3 (7.1)	4 (26.7)	4 (26.7)	7 (46.7)	0 (0.0)	0 (0.0)	0 (0.0)	0 (0.0)
	PCP	**36 (85.7)**	4 (9.5)	0 (0.0)	2 (4.8)	3 (50.0)	2 (33.3)	0 (0.0)	1 (16.7)	0 (0.0)	0 (0.0)	0 (0.0)
DT	SGS-HSR	**29 (69.0)**	8 (19.0)	4 (9.5)	1 (2.4)	3 (23.1)	1 (7.7)	9 (69.2)	0 (0.0)	0 (0.0)	0 (0.0)	0 (0.0)
	SGS-VHSR	**36 (85.7)**	6 (14.3)	0 (0.0)	0 (0.0)	1 (16.7)	0 (0.0)	5 (83.3)	0 (0.0)	0 (0.0)	0 (0.0)	0 (0.0)
	SFS	**32 (76.2)**	6 (14.3)	2 (4.8)	2 (4.8)	4 (40.0)	1 (10.0)	5 (50.0)	0 (0.0)	0 (0.0)	0 (0.0)	0 (0.0)
ST	OS (>100 m^2^)	**29 (69.0)**	8 (19.0)	2 (4.8)	3 (7.1)	0 (0.0)	0 (0.0)	13 (100.0)	0 (0.0)	0 (0.0)	0 (0.0)	0 (0.0)
	PGS-HSR	**25 (59.5)**	9 (21.4)	6 (14.3)	2 (4.8)	0 (0.0)	0 (0.0)	17 (100.0)	0 (0.0)	0 (0.0)	0 (0.0)	0 (0.0)
	PGS-VHSR	**34 (81.0)**	6 (14.3)	0 (0.0)	2 (4.8)	0 (0.0)	0 (0.0)	8 (100.0)	0 (0.0)	0 (0.0)	0 (0.0)	0 (0.0)
	PGS-ACC/DEC	**27 (64.3)**	8 (19.0)	6 (14.3)	1 (2.4)	0 (0.0)	0 (0.0)	14 (93.3)	0 (0.0)	0 (0.0)	0 (0.0)	1 (6.7)
CT	OTS (> 100 m^2^)	19 (45.2)	5 (11.9)	10 (23.8)	8 (19.0)	0 (0.0)	0 (0.0)	22 (95.7)	0 (0.0)	1 (4.3)	0 (0.0)	0 (0.0)
	GGS-HSR	**23 (54.8)**	6 (14.3)	9 (21.4)	4 (9.5)	1 (5.3)	0 (0.0)	18 (94.7)	0 (0.0)	0 (0.0)	0 (0.0)	0 (0.0)
	GGS-VHSR	**30 (71.4)**	6 (14.3)	3 (7.1)	3 (7.1)	0 (0.0)	0 (0.0)	12 (100.0)	0 (0.0)	0 (0.0)	0 (0.0)	0 (0.0)
	GGS-ACC/DEC	**27 (64.3)**	4 (9.5)	7 (16.7)	4 (9.5)	0 (0.0)	0 (0.0)	14 (93.3)	1 (6.7)	0 (0.0)	0 (0.0)	0 (0.0)
	GS-WCS	**40 (95.2)**	2 (4.8)	0 (0.0)	0 (0.0)	0 (0.0)	0 (0.0)	2 (100.0)	0 (0.0)	0 (0.0)	0 (0.0)	0 (0.0)

Bold numbers represent frequencies > 50%. ACC/DEC = situations with high intensity and volume of accelerations and decelerations (+2.5 m/s^2^); CON = high load/volume concentric exercises (>80% RM); CT = competitive tasks; DT = directed tasks; ECC = high-load eccentric exercises (external devices); GGS-ACC/DEC = global game situations with high frequency of accelerations and decelerations (+2.5 m/s^2^); GGS-HSR = global game situations with high-speed running (20–24 km/h); GGS-VHSR = global game situations with very high-speed running (>24 km/h) or >80% V_max_; GS-WCS = game situations reproducing or exceeding the demands of the most demanding scenarios (WCS); GT = general tasks; H-ECC = hip-dominant eccentric exercises; HIT = high-intensity training; HSR = high-speed running (>20 km/h); IPC = isometric posterior chain exercises; K-ECC = knee-dominant eccentric exercises; OS (>100 m^2^) = opposing situations where the relative area per player is greater than 100 m^2^; OTS (>100 m^2^) = opposing tactical situations where the relative area per player exceeds 100 m^2^; PCP = high-demand posterior chain plyometric exercises; PGS-ACC/DEC = partial game situations with high frequency of high-intensity accelerations and decelerations (+2.5 m/s^2^); PGS-HSR = partial game situations with high-speed running (20–24 km/h); PGS-VHSR = partial game situations with very high-speed running (>24 km/h) or > 80% V_max_; RS = resisted-sprint training; RST = repeated-sprint training; SIT = sprint interval training; SFS = specific force situations applied to the game; SGS-HSR = simplified game situations with high-speed running (20–24 km/h); SGS-VHSR = simplified game situations with very high-speed running (>24 km/h); ST = special tasks.

## Data Availability

The original contributions presented in this descriptive study are included in the article/Supplementary Material. Further inquiries can be directed to the corresponding author.

## Supplementary Materials

Supplementary material can be found at: https://doi.org/10.6084/m9.figshare.24975015.v1.
